# Rare case of Fungal Endocarditis secondary to a perforated diverticulum

**DOI:** 10.1186/1749-8090-10-S1-A31

**Published:** 2015-12-16

**Authors:** Asif Masroor Farooqui, Emily Hart, Onyekwelu Nzewi

**Affiliations:** 1 Royal Victoria Hospital Cardiothoracic Surgery Department Belfast, Belfast, Northern Ireland, UK

## Background/Introduction

Fungal endocarditis (FE) is a subtype of Infective Endocarditis (IE) which although rare carries a high mortality rate of 56% despite aggressive treatment (2). It manifests clinically with pyrexia, changing or new heart murmurs, dyspnoea, malaise, heart failure, peripheral emboli, finger clubbing and the classical textbook signs.

## Aims/Objectives

In the past, the diagnosis was often not made until post-mortem examination. Several factors positively and negatively impact our ability to diagnose FE. Because of the rarity of IFE and the non-specific symptoms there is often a low index of suspicion for it. Blood cultures are less sensitive for fungi and commonly negative. However, the large vegetations that characterize this illness seem to increase the sensitivity of TTE. Additionally, because fungal vegetations tend to be large they are more likely to embolise leading to systemic complications.

## Method

This patient was a 75 year old man with a background history of hypertension, hypercholesterolaemia, Type 2 Diabetes Mellitus, left nephroureterectomy, COPD and a PPM for complete heart block. He presented to hospital a year ago with a perforated diverticulum. He underwent colectomy and loop ileostomy however he had a stormy post-operative course.

## Results

In terms of management, the Mayo Clinic has published recommendations when device related endocarditis is suspected. These include that TOE rather than TTE should be carried out when blood cultures are positive, as there is superiority of specificity, 90% versus 30% respectively. Expeditious removal of hardware during an episode of cardiac device related endocarditis is essential to the overall successful management of this serious problem. Additionally these patients need prolonged treatment with IV antifungals guided by sensitivities, presence or absence of positive blood cultures and clinical condition.

## Discussion/Conclusion

In this unique case the patient's IFE was secondary to a perforated diverticulum eight months previously at which time Candida Albicans was first isolated. As a portal of entry for Candida, a perforated bowel is rare but not unheard of. In conclusion, combined medical and surgical management are fundamental to achieving a successful outcome for this condition, to prevent complications such as embolisation and sepsis.

## Consent

Written informed consent was obtained from the patient for publication of this abstract and any accompanying images. A copy of the written consent is available for review by the Editor of this journal

